# Prevalence and factors associated with renal dysfunction in children admitted to two hospitals in northwestern Tanzania

**DOI:** 10.1186/s12882-019-1254-9

**Published:** 2019-03-05

**Authors:** Neema Chami, Rogatus Kabyemera, Tulla Masoza, Emmanuela Ambrose, Franscisca Kimaro, Neema Kayange, Adolfine Hokororo, Francis F. Furia, Rob Peck

**Affiliations:** 1 Department of Pediatrics and Child Health, Bugando Medical Centre, P. O. Box 1370, Mwanza, Tanzania; 2 Department of Pediatrics and Child Health, Catholic University of Health and Allied Sciences-Bugando, P. O. Box 1464, Mwanza, Tanzania; 3 Department of Pediatrics and Child Health, College of Health Sciences-University of Dodoma, P. O. Box 395, Dodoma, Tanzania; 4 Department of Pediatrics and Child Health, Muhimbili University of Health and Allied Sciences, P. O. Box 65001, Dar es salaam, Tanzania

**Keywords:** Renal dysfunction, Estimated glomerular filtration rate (e-GFR) and serum creatinine

## Abstract

**Background:**

It is evident that renal dysfunction (RD) is associated with unique infectious and non-infectious causes in African children. However, little data exists about the prevalence and factors associated with RD in children admitted to African hospitals.

**Methods:**

In this cross-sectional study, we enrolled all children admitted to pediatric wards of Bugando Medical Centre (BMC) and Sekou-Toure Regional Referral hospital (SRRH) during a 6 month time period. Socio-demographical, clinical and laboratory data were collected using a structured questionnaire. Estimated glomerular filtration rate (eGFR) was calculated using modified Schwartz equation and those with < 60 ml/min/1.73m^2^were considered to have RD. Data analysis was done using STATA version 13 and considered significant when *p*-value was < 0.05.

**Results:**

A total of 513 children were enrolled, of which 297 (57.9%) were males. Median age of children with and without RD was 34 months (27–60) and 46.5 (29–72) respectively. Prevalence of RD was 16.2%. Factors associated with RD were herbal medication use (*p* = 0.007), history of sore throat or skin infection (*p* = 0.024), sickle cell disease (SCD) (*p* = 0.006), dehydration (*p* = 0.001), malaria (*p* = 0.01) and proteinuria (*p* = < 0.001).

**Conclusions:**

High prevalence of RD was observed among children admitted to referral hospitals in Mwanza. Screening for RD should be performed on admitted children, particularly those with history of herbal medication use, sore throat/skin infection, SCD, dehydration and malaria. Where creatinine measurement is not possible, screening for proteinuria is a reasonable alternative.

## Background

Renal dysfunction (RD) in children is still a major health problem with unclear epidemiology especially in resource limited settings [1, 2]. Clinicians treating children with RD in these settings, face numerous challenges and therefore a better understanding of RD is needed [3–5]. The prevalence of impaired renal function is estimated to range between 10 and 20% among adult population [4]. However, a notable prevalence of 13.5% among children admitted due to different causes was previously reported [6, 7]. Owing to several factors, the diagnosis and burden of RD in children differs from place to place in the world [8].

Due to lack of national registries, reported incidence and prevalence of RD from developing countries are mainly based only on reports from tertiary centers, depending on local referral system and accessibility to care [9, 10]. The extent of RD among children in Tanzania is difficult to ascertain due to challenges in diagnosis and recording. Recent studies done in our country showed a higher prevalence of renal disease among selected children population [11–14]. In northwest Tanzanian referral hospitals, the precise prevalence of RD among hospitalized children is not known. However, 44.3 and 28.8% of pediatric Human Immunodificiency Virus (HIV) infected outpatients in Mwanza and Kilimanjaro Christian Medical College (KCMC) respectively had evidence of renal dysfunction, diagnosed either by decreased eGFR or albuminuria [12, 14]. Factors associated with RD in children that were previously studied in our setting include HIV infection, schistosomiasis and sickle cell disease (SCD). We therefore conducted this study in order to determine the prevalence and more factors which are associated with RD among children admitted to pediatric wards of two referral hospitals in northwestern Tanzania. This study was conducted in a Schistosomiasis high endemic area and we therefore hypothesized that the prevalence of RD among admitted children would be high. Among other factors which might contribute to high prevalence is use of herbal medicine which is common in Tanzania [15].

## Methods

### Study site

This hospital-based cross-sectional study was conducted among children admitted to Bugando Medical Centre (BMC) and Sekou-Toure Regional Referral (SRRH) hospitals based in Mwanza – Tanzania, between August 2014 and February 2015. BMC is a tertiary hospital and serves as the zonal referral hospital and a university teaching hospital for the Catholic University of Health and Allied Sciences (CUHAS). SRRH on the other hand, is the regional referral hospital and serves patients from Mwanza region and its districts. Both two hospitals have pediatric wards which admit children aged 0–12 years. On average, 5–10 children are admitted daily to these 2 pediatric wards.

### Study population

All children aged 2–12 years with an index admission to pediatric wards at BMC and SRRH during the study period were evaluated for possible study participation. Children < 2 years old were excluded because the equation used for estimating the glomerular filtration rate was not accurate below this age group [4, 5]. Patients who died before obtaining blood sample for serum creatinine measurement were also excluded. All remaining children aged 2–12 years, admitted to the pediatric general wards at BMC and SRRH, were included after obtaining consent from their parents or guardians.

### Study procedures

Both clinical and laboratory data were collected from participants by research assistants under supervision of principal investigator within 12 h of admission. Parents and caregivers of recruited children had a face-to-face interview with the investigator. Modified version of the World Health Organization (WHO) STEPwise Approach to Surveillance (STEPS), recommended by WHO for determining the prevalence of non-communicable diseases and their risk factors, was used in this study. The sample size was calculated using Kish Leslie formula whereby a prevalence of 50% was subjected to the formula, giving a minimum sample size of 384 patients. Children were enrolled daily as they were admitted during the study period until the desired sample size was reached.

Each participant was examined thoroughly and measurements of height/length, weight and blood pressure were also taken. Height was measured while the child was standing on the measuring board stadiometer for children who were able to stand, while length board was used for children who were seriously ill and those not able to stand. Weight was measured using a DETECTO scale (WEB CITY, U.S.A), which was adjusted to zero before each measurement. Children’s weight was recorded to the nearest 100 g. The body mass index (BMI) = weight (kg)/ height^2^ (m^2^) was calculated for those above 5 years and interpreted according to WHO body mass index for age charts.

Blood pressure (BP) measurements were taken three times using a manual cuff and a sphygmomanometer. The measurements were taken twice on the right arm of a relaxed child 5 minutes apart, and once on the left arm. The reading was recorded as an average of the three readings, and interpreted as high BP whenever systolic blood pressure (SBP) or diastolic blood pressure (DBP) was >90th centile for age and height [16].

All participants with unknown HIV status were tested under provider initiated testing and counseling (PITC) using Rapid HIV antibody test as recommended by WHO and current policy of the Ministry of Health and Social Welfare (MoHSW) of Tanzania [17]. Determine HIV1/2 (Alere Medical Co. Ltd., Japan) test was used as the first test followed by Unigold (Trinity Biotech Plc, Bray, Ireland) as the second antibody test. No discordant HIV test result was noted.

Random blood glucose was checked for each participant using a ONE TOUCH glucometer (LifeScan, Inc., Milpitas California, USA). The skin of the index finger was cleaned by using a 70% isopropyl alcohol swab and pricked to obtain blood drop for testing. Blood for making thick blood smear (BS) for malaria testing was obtained from the same prick. The first drop was wiped away using clean gauze and the finger was squeezed gently to get the second blood drop from which two slides were made. Samples were then sent to the laboratory to be processed and read by an experienced laboratory technician.

Fresh mid-stream, clean catch urine specimens were collected using clean containers at admission, and were tested for proteinuria and red blood cells using MultistixTM (Healgen, TX, USA) and albuminuria using Chemstrip Micral (Roche, Mannheim, Germany). The test strip was immersed in a urine sample for 5 s, and then placed on a surface of a collection cup in order to drain excess urine. Reading was done after approximately 1 min. The color of the test pad was matched with the color scale on the vial containing the test strips. For albuminuria, the results were interpreted as “0” if negative, “1”if 20 mg/l, “2”if 50 mg/l and “3”if 100 mg/l. The Multistix TM test for proteinuria and other parameters was performed using almost similar principles.

Urine circulating cathodic antigen (CCA) [Rapid Medical Diagnostics, Pretoria, South Africa] was performed to detect schistosomal antigen. This test can detect both *S. haematobium* and *S. mansoni*species with higher sensitivity for *S. mansoni* infection. CCA is an immune-chromatographic dipstick, where the intensity of reaction band is visually rated against a reference aid. Any visible line on the test was considered positive, interpreted as, “0” if no line was visible on the test side, “1” if test line faintly visible, “2” if test line visible but lighter than control, “3” if test line’s intensity equal to control, “4” if test line darker than control as per manufacturer guidelines.

Two millilitres of blood specimen was obtained from each participant from cubital vein after cleaning with 70% alcohol swab then left to dry, followed by aseptic puncture of the vein. The specimen was collected in a syringe and then transferred to a labeled plain vacuum tube. Blood specimens were sent to the laboratory for analysis and were stored at 2-8 °C for bulky analysis to determine serum creatinine using a COBAS INTEGRA, 400 Plus machine (Roche, Germany), employing the buffered kinetic Jaffe’ reaction. Serum creatinine was Integrated Database Management System (IDMS) traceable and was measured once. The results of serum creatinine obtained were used to calculate the eGFR using a modified pediatric Schwartz equation as follows: eGFR = (k * height)/ SCr where k = 0.413, height (cm) and serum creatinine in mg/dl. Renal dysfunction was defined as eGFR< 60 ml/min/1.73m^2^. It was not possible to categorize RD using acute kidney injury (AKI) criteria or chronic kidney disease (CKD) as baseline creatinine was not known and 3 months duration had not been attained for CKD [11, 12].

### Data analysis

Microsoft Excel was used for data coding and cleaning after entry. Data was transferred to software for analysis using STATA version 13 (College Station, Texas, USA). The primary endpoint of the study was the prevalence of renal dysfunction, predefined as eGFR of less than 60 ml/min at admission. Independent variables were age, gender, HIV status, blood slide (BS) for Malaria parasite results, analgesic use, schistosomiasis test results, diarrhea, nutritional status, hypertension, fever/sepsis, dehydration status and reported urine output.

Continuous variables were summarized as mean with standard deviation (for normally distributed continuous variables) and median with interquartile ranges (for non-normally distributed continuous variables). We used probability plots and Shapiro-Wilk normality test to assess the normality of distribution of continuous variables. Categorical variables were summarized as proportions or percentages. The prevalence of kidney dysfunction was determined by taking the number of children with the disease over the enrolment number. Factors associated with RD were determined by univariate and multivariate logistic regression with 95% confidence intervals reported.

## Results

### Participants’ enrollment and characteristics

**Fig. 1:**
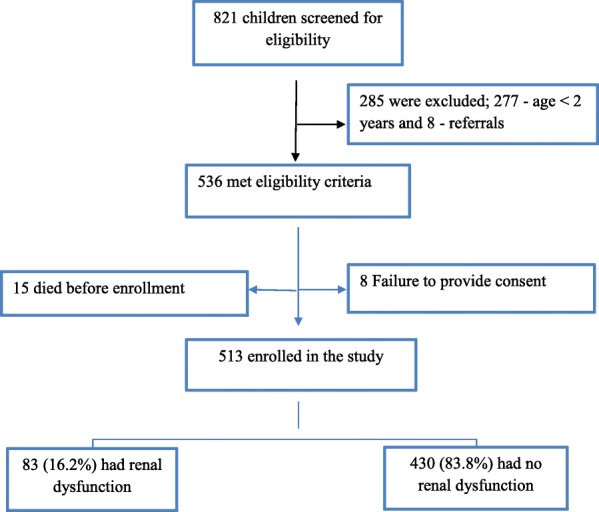
Study enrollment overview

A total of 821 children were seen between August 2014 and February 2015. A total of 285 (34.7%) children were excluded because 277 (97.2%) were aged less than 2 years and 8 (2.8%) were referred cases from other hospitals. Five hundred and thirty six children (65.2%) children, admitted to the respective hospitals were eligible for recruitment out of which 8 (1.5%) children were excluded because they failed to provide consent and 15 (2.8%) died before enrollment. Only 513 (95.7%) were included in the final analysis (Fig. 1). Among the study participants, there was a slight male preponderance of 297 (57.9%) and all children were of African ethnicity. The majority, 369 (71.9%), were admitted to BMC. One-third, 163 (31.8%), were using either lake or pond water for their daily activities (Table 1).

**Table 1:** Baseline socio-demographic, clinical and laboratory characteristics of studyparticipants

Factor	Median/IQR, n (%) *N* = 513	With RD *N* = 83	Without RD *N* = 430	Univariate OR [95% CI]	*p*-value	Multivariate OR [95% CI]	*p*-value
Female gender	216 (42.1)	41 (49.4)	175 (40.7)	1.42 [0.89–2.28]	0.14		
Age in months	42 [28–72]	34 [27–60]	46.5 [29–72]	0.99 [0.99–1.00]	0.10		
Study site							
Bugando Medical Centre	369 (71.9)	65 (78.3)	304 (70.7)	RG0.67	RG		
Sekou Toure Hospital	144 (28.1)	18 (21.7)	126 (29.3)	[0.38–1.17]	0.16		
Water source							
Tap water	350 (68.2)	58 (69.9)	292 (67.9)	0.91 [0.55–1.52]	0.72		
Lake or pond water	163 (31.8)	25 (30.1)	138 (32.1)				
Toilet							
Modern flush toilet	250 (48.7)	35 (42.2)	215 (50.0)	1.37 [0.85–2.21]	0.19		
Pit latrine	263 (51.3)	48 (57.8)	215 (50.0)				
Medical history							
Herbal medications use	187 (36.5)	45 (54.2)	142 (33.0)	2.40 [1.50–3.87]	< 0.001	2.11 [1.23–3.61]	0.007
Gentamycin use	47 (9.2)	7 (8.4)	40 (9.3)	0.90 [0.34–2.08]	0.80		
Ceftriaxone use	51 (9.9)	8 (9.6)	43 (10.0)	0.96 [0.43–2.12]	0.92		
NSAID use	81 (15.8)	10 (12.1)	71 (16.5)	0.69 [0.34–1.41]	0.31		
Throat/skin infection	36 (7.0)	11 (13.3)	25 (5.8)	2.48 [1.17–5.25]	0.018	2.74 [1.14–6.56]	0.024
History of SCD	71 (13.8)	21 (25.3)	50 (11.6)	2.5 [1.44–4.58]	0.001	2.59 [1.31–5.10]	0.006
Family history of SCD	40 (7.8)	6 (7.2)	34 (7.9)	0.91 [0.37–2.24]	0.83		
Prior surgeries	28 (5.5)	1 (1.2)	27 (6.3)	0.18 [0.02–1.36]	0.10		
Presenting symptoms							
Hematuria	28 (5.5)	4 (4.8)	24 (5.6)	0.86 [0.29–2.54]	0.78		
Fever	373 (72.7)	59 (71.1)	314 (73.0)	0.91 [0.54–1.53]	0.72		
Dysuria	63 (12.3)	12 (14.5)	51 (11.9)	1.26 [0.64–2.48]	0.51		
Rash	28 (5.5)	6 (7.2)	22 (5.1)	1.45 [0.57–3.68]	0.44		
Diarrhea	112 (21.8)	23 (27.7)	89 (20.7)	1.47 [0.86–2.51]	0.16		
Bloody diarrhea	24 (4.7)	7 (8.4)	17 (4.0)	2.24 [0.90–5.58]	0.08		
Vomiting	176 (34.3)	27 (32.5)	149 (34.7)	0.91 [0.55–1.50]	0.71		
Recurrent UTI	45 (8.8)	5 (6.0)	40 (9.3)	0.63 [0.24–1.63]	0.34		
Urine output							
Normal	458 (89.3)	66 (79.5)	392 (91.2)	RG	RG		
Increased	15 (2.9)	2 (2.4)	13 (3.0)	0.91 [0.20–4.14]	0.91		
Decreased	40 (7.8)	15 (18.1)	25 (5.8)	3.56 [1.79–7.11]	< 0.001	1.11 [0.60–2.06]	0.73
Presenting signs							
Pallor	212 (41.3)	32 (38.6)	180 (41.9)	0.87 [0.54–1.41]	0.58		0.001 0.62
Dehydration	65 (12.7)	21 (25.3)	44 (10.2)	2.97 [1.66–5.33]	< 0.001	3.12 [1.56–6.26]	
Edema	59 (11.5)	15 (18.1)	44 (10.2)	1.94 [1.02–3.67]	0.04	1.21 [0.56–2.62]	
Vital signs							
Pediatric GCS	15 [15–15]	15 [15–15]	15 [15–15]	0.83 [0.69–0.10]	0.05		
Saturation	97 (95–98)	97 [94–98]	97 [95–98]	0.97 [0.93–1.01]	0.09		
Temperature	37 [36.6–38]	36.5 [36.2–36.8]	37 [36.6–38]	0.92 [0.72–1.16]	0.47		
Respiratory rate	30 [23–40]	30 [24–40]	30 [22–38]	1.01 [0.10–1.03]	0.08		
Pulse rate	112 [98–128]	110 [98–128]	112 [98–130]	0.10 [0.99–1.01]	0.38		
Blood pressure							
SBP	90 [85–100]	90 [85–102]	90 [85–100]	1.01 [0.99–1.03]	0.27		
DBP	60 [58–67]	60 [60–70]	60 [58–66]	1.01 [0.99–1.04]	0.29		
HIV positivity	30 (5.8)	5 (6.0)	25 (5.8)	1.04 [0.38–2.79]	0.94		
Positive BS for MPS	59 (11.5)	16 (19.3)	43 (10.0)	2.15 [1.14–4.03]	0.017	2.67 [1.26–5.66]	0.01
Urine protein							
Negative	417 (81.3)	46 (55.4)	371 (86.3)	RG	RG		
Positive	96 (18.7)	37 (44.6)	59 (13.7)	5.06 [3.03–8.45]	< 0.001	5.28 [2.97–9.39]	< 0.001
Urine RBC							
Negative	493 (96.1)	77 (92.8)	416 (96.7)	RG	RG		
Positive	20 (3.9)	6 (7.2)	14 (3.3)	2.32 [0.86–6.21]	0.10		
Urine CCA							
Negative	344 (67.1)	47 (56.6)	297 (69.1)	RG	RG		
Positive	169 (32.9)	36 (43.4)	133 (30.9)	1.71 [1.05–2.76]	0.028	1.19 [0.36–3.95]	0.77
Urine CCA							
Negative	344 (67.1)	47 (56.6)	297 (69.1)	RG	RG		
Line faintly visible	83 (16.2)	19 (22.9)	64 (14.9)	1.88 [1.03–3.41]	0.039	1.31 [0.36–4.80]	0.68
Line lighter than control	64 (12.5)	13 (15.7)	51 (11.9)	1.61 [0.81–3.41]	0.17		
Intensity equal to control	22 (4.2)	4 (4.8)	18 (4.2)	1.40 [0.46–4.33]	0.56		
Nutritional status							
Normal	268 (52.2)	47 (56.6)	221 (51.4)	RG	RG		
Mild malnutrition	95 (18.5)	12 (14.5)	83 (19.3)	0.68 [0.34–1.34]	0.23		
Moderate malnutrition	76 (14.8)	12 (14.5)	64 (14.9)	0.88 [0.44–1.76]	0.72		
Severe malnutrition	74 (14.5)	12 (14.5)	62 (14.4)	0.91 [0.45–1.82]	0.79		

*CI* Confidence interval, *RG* Reference group, *BS for MPS* Blood Slide for Malaria Parasites, *NSAD* Non-Steroidal Anti-inflammatory Drugs, *SCD* Sickle Cell Disease, *UTI* Urinary Tract Infection, *GCS* Glasgow Coma Score, *RR* Respiratory Rate, *SBP* Systolic Blood Pressure, *DBP* Diastolic Blood Pressure, *HIV* Human Immunodeficiency Virus, CCA Circulating Cathodic Antigen

### Prevalence and characteristics of participants with and without renal dysfunction

The overall prevalence of RD among admitted patients in the two referral hospitals was 16.2% (83 participants). The median age of children with RD was 34 (27–60) months while that of children without RD was 46 (29–72) months. When aggregated by sex, the proportion of children with RD in both sexes was equal. Among those with RD, 48 (57.8%) used pit latrine at their home and water source was the lake/ponds for 25 (30.1%) participants (Table 1).

Out of the 513 children, 187 (36.5%) had a history of herbal medication use, of which 45 (24.1%) had RD. Only 4 children had been previously diagnosed with renal disease: 2 had nephrotic syndrome and the other two had nephroblastoma as the primary diagnosis on admission. In addition, 71 (13.8%) had sickle cell disease and 21 (25.3%) of these had RD. Among those with RD, 11 (13.3%) had a previous history of sore throat or skin infection.

The common presenting symptoms reported by the study participants were fever-373 (72.7%), vomiting-176 (34.3%) and diarrhea-112 (21.8%). Some features of kidney disease like hematuria and dysuria were relatively uncommon, accounting for 28 (5.5%) and 63 (12.3%) of cases respectively. Fifteen children (2.9%) reported to have increased urine output (polyuria) and 40 (7.8%) had a decreased urine output. However, the majority had normal urine output (89.3%) as reported by parents/ caretakers. Nearly half of the study subjects, 245 (47.8%), had malnutrition. Most children had a normal systolic and diastolic blood pressure percentile for age and height. Two hundred and twelve children (41.3%) presented with pallor of varying degrees and almost all children had normal Glasgow coma score at admission, with stable vital signs as described in Table 1.

### Factors associated with renal dysfunction

All presenting demographic and clinical characteristics were evaluated as possible factors predictive of renal dysfunction. Significant predictors of RD on univariate analysis included: herbal medication use (OR = 2.40, 95%CI 1.50–3.87, *p* < 0.001), throat/skin infection (OR = 2.48, 95%CI 1.17–5.25, *p* = 0.018), history of SCD (OR = 2.5, 95%CI 1.44–4.58, *p* = 0.001), decreased urine output (OR = 3.56, 95%CI 1.79–7.11, *p* = < 0.001), dehydration (OR = 2.97, 95%CI 1.66–5.33, *p* = < 0.001) and presence of edema (OR = 1.94, 95%CI 1.02–3.67, *p* = 0.04). Children who had a positive blood slide for malaria parasites on admission were noted to have two fold increased likelihood of having with renal dysfunction (OR = 2.15, 95%CI 1.14–4.03, *p* = 0.017). The presence of Schistosomiasis was also significantly associated with renal dysfunction (OR = 1.71 95%CI 1.05–2.76, *p* = 0.028). In multivariate analysis, factors that remained significant included: herbal medications use (OR = 2.11, 95%CI 1.23–3.61, *p* = 0.007), history of throat/skin infection (OR = 2.74 95%CI 1.14–6.56, *p* = 0.024), history of SCD (OR = 2.59, 95%CI 1.31–5.10, *p* = 0.006), dehydration (OR = 3.12, 95%CI 1.56–6.26, *p* = 0.001), positive blood slide for malaria parasites (OR = 2.67, 95%CI 1.26–5.66, p = 0.01) and proteinuria (OR = 5.28, 95%CI 2.97–9.39, *p* = < 0.001) (Table 1).

## Discussion

This study was carried out to determine the prevalence, clinical characteristics and factors associated with renal dysfunction in hospitalized children in Tanzania. The prevalence of RD among admitted children was 16.2% which is higher than previous studies done among outpatient HIV infected children in Dar-es-salaam (5.8%) and Mwanza (7.4%) [11, 12]. The same study done in Mwanza-Tanzania, reported 4.9% prevalence of RD in HIV uninfected children [12]. Though HIV infection did not predict for RD in this study, the observed difference could be explained by the fact that children in our study had more co-morbid conditions which could have predisposed them to RD compared to studies done in Mwanza and Dar-es-salaam.

Our study found a significant association between herbal medications use and renal dysfunction. Half of the children with RD were using herbal medicines at the time of admission and use of herbal medicines was independently associated with a > 2-fold increased odds of RD. Previous studies demonstrated that using traditional remedies [18, 19] is highly predictive of AKI, as reported in 26% of hospitalized children and 18% in those who are in the community [19]. Herbs are also known to cause interstitial nephritis and tubule damage particularly when used for a long time causing chronic kidney disease [20]. The lower percentage of those who used traditional remedies in this study could be attributed to the fact that we relied on a reported history from the parent/guardian.

Previous history of throat or skin infection was found to be associated with renal dysfunction in the present study. Nearly 15% of children with RD reported a history of throat or skin infection and history of such infections was independently associated with a nearly 3-fold increased odds of RD. Acute glomerulonephritis has been pointed out in literature to be associated with streptococcal throat infection [21]. This has also been observed in a study done at a single hospital in Nigeria as an important association with acute glomerulonephritis (AGN) in 66.6 and 38.7% of those with AGN had the evidence of RD [22]. The lower proportion in our study can be explained by the fact that our study population included all children who were admitted for different reasons, as opposed to the Nigerian study which was conducted in children who had a risk factor for RD.

This study showed a significant association between RD and the presence of dehydration on physical examination at the time of admission. One-quarter of children with RD were clinically dehydrated on admission vs. 10% of those without RD (OR = 3.12 [1.56–6.26]). It has been shown in some studies that dehydration was the common cause of acute kidney injury among inpatients using local herbs [20, 23] and this can be attributed to synergistic effects of infections and toxicity among other factors. Some studies showed that dehydration contributed to 7.8% cases of RD [24]. The difference could be attributed to the fact that, we included all degrees of dehydration (moderate and severe) as opposed to the other study, which included only those with severe dehydration.

Having a positive sickling test (indicating sickle cell disease or trait) was also found to be a significant predictor of renal dysfunction. We demonstrated that 25.3% of children with SCD diagnosed by sickling test had RD and a positive sickling test was associated with a > 2.5-fold increased odds of RD. Previous study in sub-Saharan Africa reported a lower prevalence of SCD among children with RD and this may be explained by the fact that they used only children with confirmed sickle cell anemia (HbSS) as their study population [25]. Children with SCD are likely to have persistent proteinuria and renal insufficiency which was found in 12.3% of children suffering from SCD [25–27]. Of note, recent data indicates that sickle cell trait may also be associated with RD.

Among children diagnosed with malaria by the positive blood slide, significant association between RD and malaria was demonstrated. Approximately 19% of children with RD had positive blood slide for malaria parasites compared to 10% of children without RD (aOR = 2.67 [1.26–5.66]). The study conducted in Nigeria among admitted children found that malaria was responsible for 11.4% of acute kidney injury cases [28]. Our study had a higher percentage as compared to the Nigerian study as the two studies employed different methodologies in that the current study was a prospective one while the latter study was a retrospective one where they did case review of children admitted at a particular time. They also used modified pediatric risk, injury, failure, loss o function and end stage renal disease (RIFLE) criteria in the detection of AKI, as opposed to our study where we used modified Schwartz formula. Several other studies show correlation between malaria and RD in different countries [7, 28–31].

The presence of proteinuria was found in 44.6% of children who were found to have RD, compared to 13.7% without RD (OR = 5.06 [3.03–8.45]), a finding which may suggest renal parenchymal damage. Other studies showed a lower prevalence of proteinuria of approximately 7–26% among the study subjects. This may be attributed to the fact that, primary admission diagnoses in these studies differ from those of the present study [7, 11, 13, 23, 25–27, 31].

Our study has described factors which are associated with renal dysfunction (RD) in children and a high prevalence of RD in Tanzania. Therefore, children at risk of RD should be screened in order to provide timely intervention for RD and avoid RD related complications. However, several limitations are acknowledged. Differentiation between AKI and CKD could not be made as only single serum creatinine was measured and urine output was not measured to define acute kidney injury. Also, the duration of 3 months required to define CKD could not be ascertained. Furthermore, common febrile illnesses in our study setting may present with proteinuria, which is reversible after recovery, and therefore may not provide a true reflection of RD in children among children who had febrile illnesses. Future studies should seek to obtain baseline creatinine values in populations at high-risk for renal disease and should determine how these values change over time including with hospitalization.

## Conclusion

The prevalence of renal dysfunction among admitted children in the two-referral hospitals in Tanzania’s lake zone was 16.2%. Factors associated with renal dysfunction were herbal medication use, previous history of throat or skin infections, having sickle cell disease, dehydration on admission, malaria as detected by a positive blood smear and proteinuria. Clinical evaluation and detection of proteinuria using the urine dipstick analysis can be used to identify those who are at risk of renal dysfunction among admitted children in resource limited settings and hence refer them early for further work up. Efforts should be made to prevent the occurrence of preventable childhood diseases that predict RD.

## Abbreviations

AGN: Acute glomerulonephritis
AKI: Acute kidney injury
aOR: Adjusted odds ratio
BMC: Bugando Medical Centre
BMI: Body mass index
BP: Blood pressure
BS: Blood smear
CCA: Circulating cathodic antigen
CKD: Chronic kidney disease
CUHAS: Catholic University of Health and Allied Sciences
DBP: Diastolic blood pressure
eGFR: Estimated glomerular filtration rate
GFR: Glomerular filtration rate
HbSS: Hemoglobin SS
IDMS: Integrated Database Management System
KCMC: Kilimanjaro Christian Medical College
MoHSW: Ministry of Health and Social Welfare
OR: Odds ratio
PITC: Provider initiated testing and counseling
RD: Renal dysfunction
RIFLE: Risk, Injury, Failure, Loss of function, End stage kidney disease
SBP: Systolic blood pressure
SCD: Sickle cell disease
SRRH: Sekou-toure regional referral hospital
STEPS: STEPwise approach to surveillance
WHO: World Health Organization
